# Munc18c accelerates SNARE-dependent membrane fusion in the presence of regulatory proteins α-SNAP and NSF

**DOI:** 10.1016/j.jbc.2024.105782

**Published:** 2024-02-21

**Authors:** Furong Liu, Ruyue He, Xinyu Xu, Min Zhu, Haijia Yu, Yinghui Liu

**Affiliations:** Jiangsu Key Laboratory for Molecular and Medical Biotechnology, College of Life Sciences, Nanjing Normal University, Nanjing, China

**Keywords:** membrane fusion, SNARE, Munc18c, SM protein, α-SNAP, NSF

## Abstract

Intracellular vesicle fusion is driven by the soluble N-ethylmaleimide-sensitive factor attachment protein receptors (SNAREs) and their cofactors, including Sec1/Munc18 (SM), α-SNAP, and NSF. α-SNAP and NSF play multiple layers of regulatory roles in the SNARE assembly, disassembling the cis-SNARE complex and the prefusion SNARE complex. How SM proteins coupled with NSF and α-SNAP regulate SNARE-dependent membrane fusion remains incompletely understood. Munc18c, an SM protein involved in the exocytosis of the glucose transporter GLUT4, binds and activates target (t-) SNAREs to accelerate the fusion reaction through a SNARE-like peptide (SLP). Here, using an *in vitro* reconstituted system, we discovered that α-SNAP blocks the GLUT4 SNAREs-mediated membrane fusion. Munc18c interacts with t-SNAREs to displace α-SNAP, which overcomes the fusion inhibition. Furthermore, Munc18c shields the *trans*-SNARE complex from NSF/α-SNAP-mediated disassembly and accelerates SNARE-dependent fusion kinetics in the presence of NSF and α-SNAP. The SLP in domain 3a is indispensable in Munc18c-assisted resistance to NSF and α-SNAP. Together, our findings demonstrate that Munc18c protects the prefusion SNARE complex from α-SNAP and NSF, promoting SNARE-dependent membrane fusion through its SLP.

Membrane fusion, the final step of intracellular vesicular trafficking, is tightly regulated by the soluble N-ethylmaleimide-sensitive factor attachment protein receptors (SNAREs) and their regulators so that cargos are delivered to destination compartments accurately and timely (1, 2, 3, 4, 5, 6). The engine of the fusion machinery is the SNARE complex formed by the target (t-) SNAREs paired with the vesicle (v-) SNARE (7, 8). The zippering of the SNAREs brings the two apposed membranes into close proximity, leading to the formation of a four-helix *trans*-SNARE complex that catalyzes membrane fusion (9, 10, 11). After fusion, alpha soluble N-ethylmaleimide-sensitive factor attachment protein (α-SNAP) binds the *cis*-SNARE complex, in turn recruiting N-ethylmaleimide-sensitive factor (NSF), a hexameric AAA-family ATPase, to recycle the SNAREs for another round of fusion (1, 12, 13, 14, 15, 16, 17). On the other side, increasing evidence has shown that NSF and α-SNAP disassociate the assembling *trans*-SNARE complex to inhibit membrane fusion (18, 19, 20), raising a question of how *trans*-SNARE complex assembly proceeds in the presence of NSF and α-SNAP.

SNAREs alone can drive membrane fusion slowly *in vitro*, but physiological vesicle fusion *in vivo* requires additional regulators, including Sec1/Munc18 (SM) proteins, to modulate accurate cargo transport (21, 22). SM proteins directly interact with SNAREs in different stages. It is worth testing how SM proteins act on the fusion reactions in the presence of NSF and α-SNAP. It has been reported that SM proteins Sly1 and Vps33 shield SNARE complexes from Sec17-(α-SNAP) and Sec18-(NSF) mediated disassembly (23). Recent studies show that SM protein Munc18-1, acting in concert with Munc13 and synaptotagmin, orchestrates SNARE complex assembly in an NSF/α-SNAP-resistant manner (18, 24). It is still uncertain whether the protection of the *trans*-SNARE complex by SM protein in the presence of NSF and α-SNAP is a conserved function of SM proteins in the divergent exocytotic events.

Munc18c, a broadly distributed SM protein in mammalian cells, selectively regulates multiple exocytotic pathways, including GLUT4 exocytosis. Our previous studies suggest that Munc18c is a positive regulator that stimulates GLUT4 SNAREs-mediated vesicle fusion. Munc18c binds and activates the t-SNAREs to accelerate *trans*-SNARE zippering through its SLP in domain 3a (22, 25, 26, 27, 28). To fully address the role of Munc18c in membrane fusion, it is necessary to study whether Munc18c promotes the *trans*-SNARE assembly and membrane fusion under the regulation of α-SNAP and NSF.

In this work, we observed that α-SNAP markedly inhibits GLUT4 SNAREs-mediated membrane fusion. The addition of Munc18c reverses the fusion inhibition caused by α-SNAP and increases the fusion rate significantly. Further studies suggested that α-SNAP can be displaced from t-SNAREs by Munc18c. In addition, Munc18c resists the disassembly of the *trans*-SNARE complex, promoting efficient SNARE-mediated liposome fusion in the presence of α-SNAP and NSF. Interestingly, the resistance of Munc18c to α-SNAP or α-SNAP-NSF depends on the SLP in domain 3a. Our findings reveal that Munc18c protects the *trans*-SNARE complex from the potential NSF/α-SNAP-caused disassembly and promotes SNARE-dependent membrane fusion in the presence of NSF and α-SNAP. Orchestrating SNARE-dependent membrane fusion in an α-SNAP- and NSF-resistant manner might be a conserved mechanism for the stimulatory function of SM proteins.

## Results

### α-SNAP inhibits GLUT4 SNAREs-mediated liposome fusion

**Figure 1:**
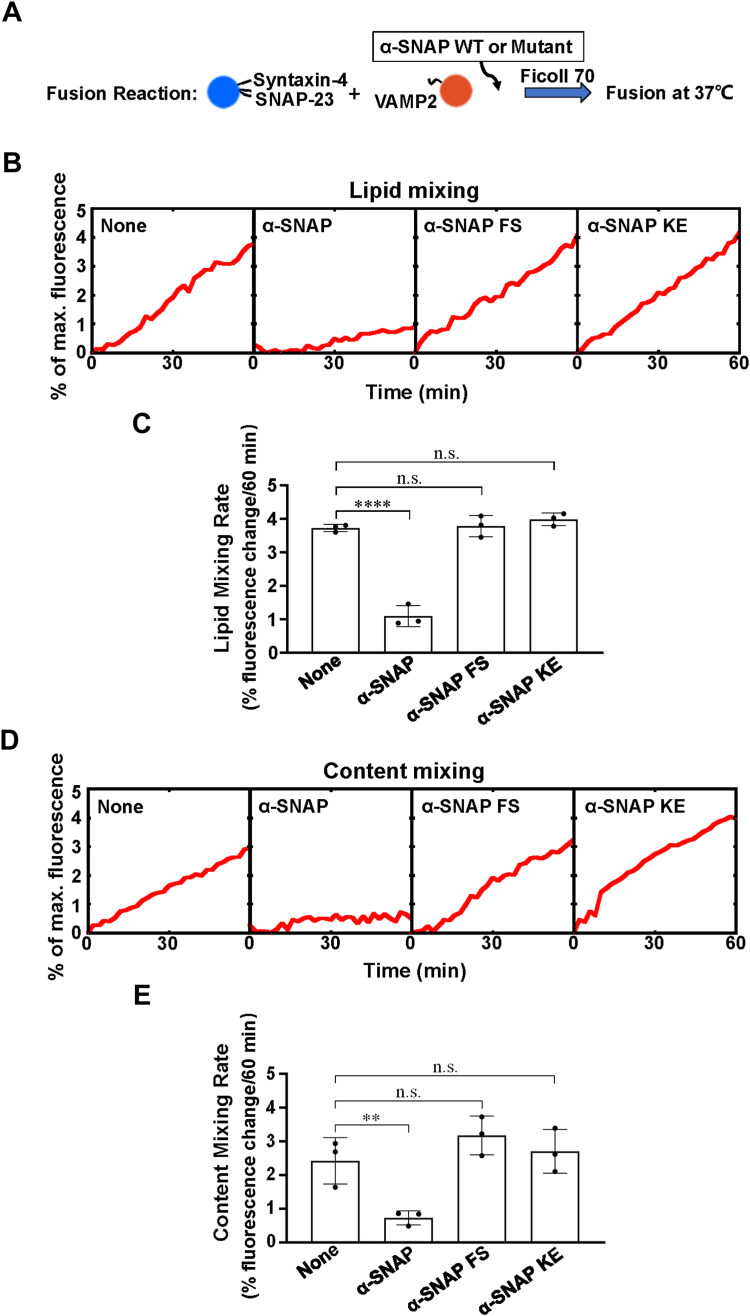
**α-SNAP inhibits GLUT4 SNARE-mediated membrane fusion with the requirement of membrane association and SNARE interaction.***A*, diagram illustrating the experimental procedure of the reconstituted fusion reactions. *B*, lipid mixing of the reconstituted fusion reactions in the absence or presence of 2 μM α-SNAP WT or Mutant proteins. Each fusion reaction contained 5 μM t-SNAREs, 1.5 μM v-SNARE, and 100 mg/ml Ficoll 70. *C*, lipid mixing rates of the reconstituted fusion reactions shown in (*B*). Data are presented as percentage of fluorescence change per 60 min. Error bars indicate standard deviation. Data are presented as mean ± SD (n = 3 independent replicates). *p* Values were calculated using ordinary one-way ANOVA with Tukey’s multiple comparisons test. n.s., *p* > 0.05. ∗∗∗∗*p* < 0.0001. *D*, content mixing of the reconstituted fusion reactions in the absence or presence of 2 μM α-SNAP WT or Mutant proteins. Each fusion reaction contained 5 μM t-SNAREs, 1.5 μM v-SNARE and 100 mg/ml Ficoll 70. *E*, content mixing rates of the reconstituted fusion reactions shown in *D*. Data are presented as percentage of fluorescence change per 60 min. Error bars indicate standard deviation. Data are presented as mean ± SD (n = 3 independent replicates). *p* Values were calculated using ordinary one-way ANOVA with Tukey’s multiple comparisons test. n.s., *p* > 0.05. ∗∗*p* < 0.01.

To study how α-SNAP acts on the GLUT4 fusion machinery independent of NSF, we employed lipid mixing and content mixing assays to monitor the fusion between v-liposomes containing VAMP2 and t-liposomes containing syntaxin-4/SNAP-23 in the presence of the macromolecular crowding agent Ficoll 70 (Fig. 1*A*) (22, 27, 29). In the fluorescence resonance energy transfer (FRET)-based lipid mixing assay, t- and v-SNAREs drove a basal level of lipid mixing, which was inhibited by wild-type (WT) α-SNAP (Figs. 1*B* and S1).

Previous studies suggested that the efficient disassembly of the SNARE complex by NSF/α-SNAP requires both the association of α-SNAP N-terminal hydrophobic loop to the membrane and the direct α-SNAP/four-helix SNARE interactions (30, 31). Two α-SNAP mutants that were previously shown to either inactivate the hydrophobic loop (30) or interfere with the SNARE binding were employed to evaluate the roles of these interactions in the fusion inhibitory activity of α-SNAP without NSF. When two adjacent phenylalanine residues in the hydrophobic loop of α-SNAP were substituted by serine (F27S/F28S, FS), the SNARE-dependent lipid mixing was no longer inhibited (Fig. 1, *B* and *C*). The mutations that were shown disrupting the α-SNAP/SNAREs interaction (K122E/K163E, KE) abrogated the inhibition of fusion kinetics (Fig. 1, *B* and *C*). In the content mixing assay, we observed that WT α-SNAP rather than α-SNAP FS or KE mutant blocked content mixing of the fusion reaction, confirming that both the intact N-terminal hydrophobic loop and SNARE binding region are critical for its inhibitory activity (Fig. 1, *D* and *E*). Together, these results demonstrate that α-SNAP alone inhibits GLUT4 SNARE-mediated membrane fusion with the requirement of membrane association and SNARE interaction.

### Munc18c overcomes the inhibition of fusion reaction by α-SNAP

**Figure 2:**
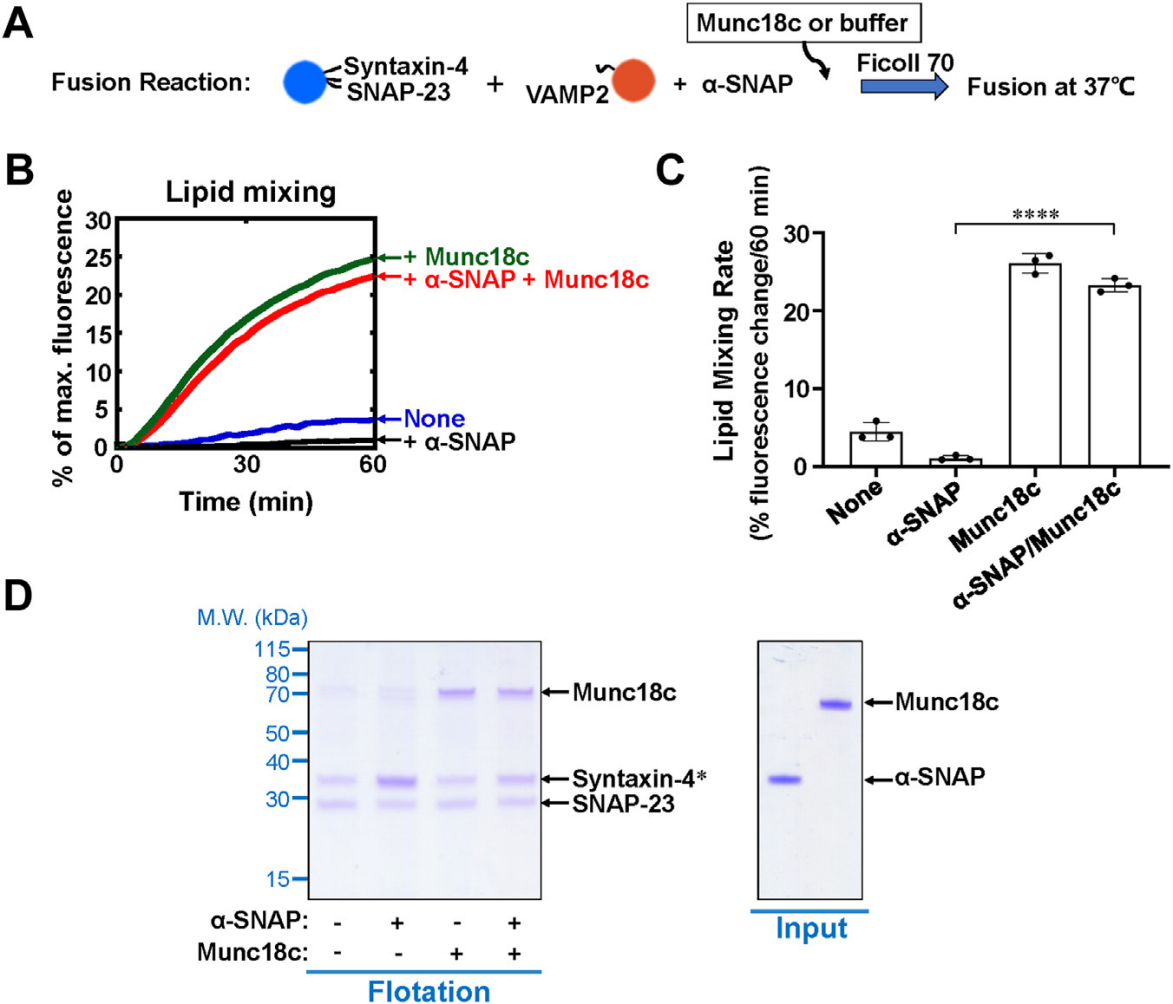
**Munc18c overcomes the inhibition of membrane fusion by α-SNAP.***A*, illustration of the reconstituted liposome fusion procedures. *B*, lipid mixing of the reconstituted fusion reactions in the absence or presence of 2 μM α-SNAP or 5 μM Munc18c. Each fusion reaction contained 5 μM t-SNAREs, 1.5 μM v-SNARE and 100 mg/ml Ficoll 70. *C*, lipid mixing rates of the reconstituted fusion reactions shown in *B*. Data are presented as percentage of fluorescence change per 60 min. Error bars indicate standard deviation. Data are presented as mean ± SD (n = 3 independent replicates). *p* Values were calculated using ordinary one-way ANOVA with Tukey’s multiple comparisons test. ∗∗∗∗*p* < 0.0001. *D*, *left*, *Coomassie blue*-stained SDS-PAGE gel showing the binding of Munc18c and α-SNAP to t-SNARE liposomes containing syntaxin-4 and SNAP-23. The liposomes were prepared with 100% phosphatidylcholine (PC). Each binding reaction contained 5 μM SNAREs and 5 μM Munc18c or α-SNAP. *Asterisk*, α-SNAP co-migrated with syntaxin-4 on SDS-PAGE but its binding to t-SNARE liposomes was evident. *Right*, *Coomassie blue*-stained SDS-PAGE gel showing recombinant α-SNAP and Munc18c proteins used in this study.

We previously reported that Munc18c, a primary SM protein in insulin-stimulated GLUT4 translocation, stimulates its cognate SNARE-mediated vesicle fusion (25, 27). Next, we explored the functional coupling between Munc18c and α-SNAP in GLUT4 SNAREs-mediated fusion reaction. In the lipid mixing experiments with Ficoll 70, Munc18c reversed the inhibition of α-SNAP on GLUT4 SNARE-dependent liposome fusion and further significantly increased the fusion rate (Figs. 2, *A*–*C* and S1). Since both α-SNAP and Munc18c bind to the t-SNAREs (12, 14), we conducted a liposome co-flotation assay to explore whether α-SNAP and Munc18c simultaneously bind to t-SNAREs or in a mutually exclusive manner. We observed that Munc18c efficiently displaced α-SNAP from syntaxin-4/SNAP-23, whereas α-SNAP could hardly disrupt the binding of Munc18c to t-SNAREs (Figs. 2*D* and S2). These data suggest that Munc18c overcomes the inhibition of α-SNAP on SNARE-dependent liposome fusion through the competitive binding to t-SNAREs.

### Munc18c promotes SNARE-dependent fusion in the presence of NSF and α-SNAP

**Figure 3:**
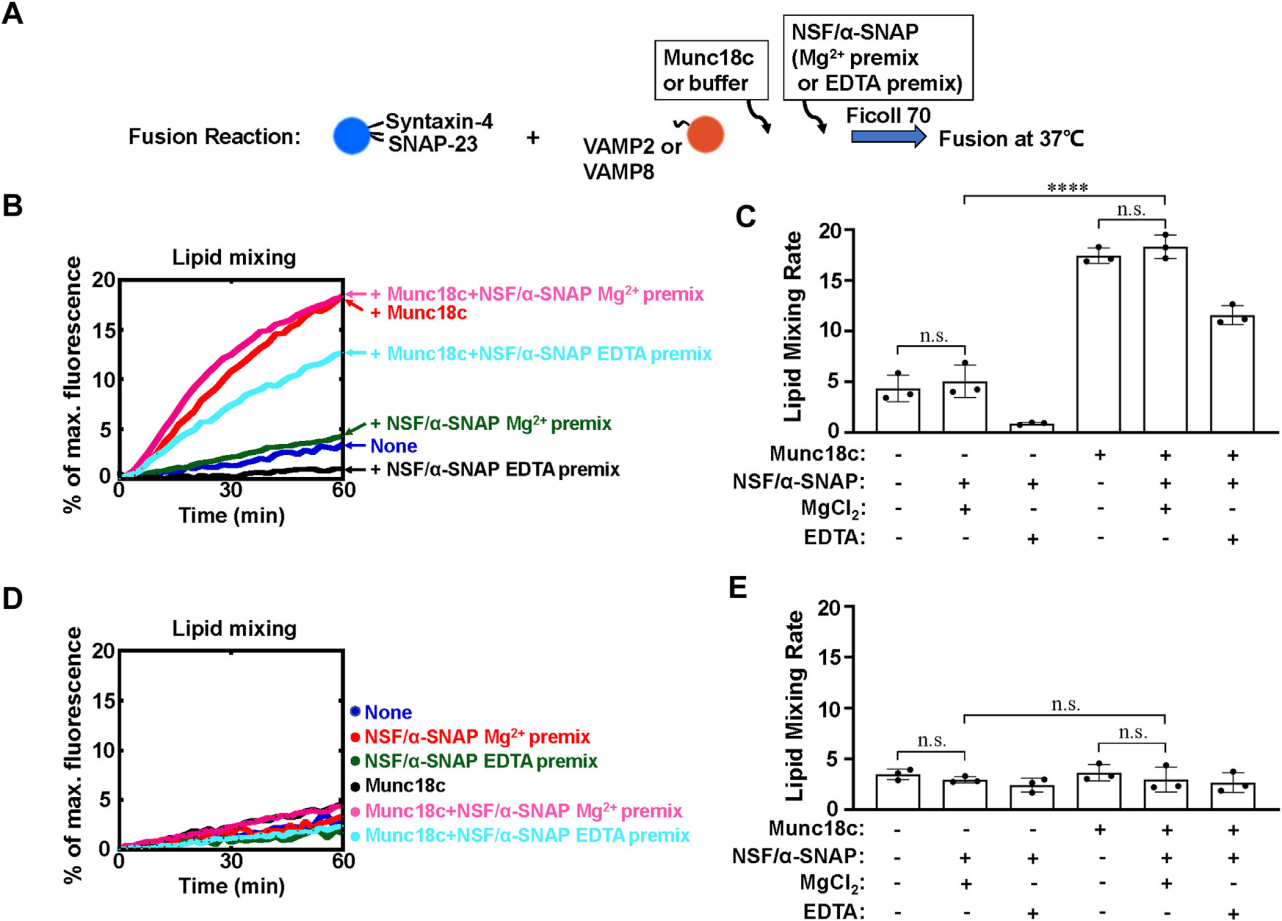
**Munc18c promotes SNARE-dependent membrane fusion in the presence of NSF and α-SNAP.***A*, illustration of the experimental procedure of the reconstituted fusion reactions. *B*, lipid mixing of the reconstituted fusion reactions shown in *A*. VAMP2 was used as the v-SNARE in the reactions. Lipid mixing assays were performed in the presence of 1 μM NSF, 2 μM α-SNAP, 2.5 mM ATP, and 5 mM MgCl_2_ (Mg^2+^ premix)/EDTA (EDTA premix) without or with 5 μM Munc18c. Each fusion reaction contained 5 μM t-SNAREs, 1.5 μM v-SNARE and 100 mg/ml Ficoll 70. *C*, lipid mixing rates of the reconstituted fusion reactions shown in *B*. Data are presented as the percentage of fluorescence change per 60 min. Error bars indicate standard deviation. Data are presented as mean ± SD (n = 3 independent replicates). *D*, lipid mixing of the reconstituted fusion reactions shown in (*A*). VAMP8 was used as the v-SNARE in the reactions. Lipid mixing assays were performed in the presence of 1 μM NSF, 2 μM α-SNAP, 2.5 mM ATP, and 5 mM MgCl_2_ (Mg^2+^ premix)/EDTA (EDTA premix) without or with 5 μM Munc18c. Each fusion reaction contained 5 μM t-SNAREs, 1.5 μM v-SNARE and 100 mg/ml Ficoll 70. *E*, lipid mixing rates of the reconstituted fusion reactions shown in *D*. Data are presented as the percentage of fluorescence change per 60 min. Error bars indicate standard deviation. Data are presented as mean ± SD (n = 3 independent replicates). *p* Values were calculated using two-way ANOVA with Tukey’s multiple comparisons test. n.s., *p* > 0.05. ∗∗∗∗*p* < 0.0001.

NSF and α-SNAP are the protein machines for disassembling SNARE complexes in eukaryotic cells. α-SNAP binds to the SNARE complex and recruits NSF, leading to the disintegration of the SNARE complex. When NSF was included, the inhibition of α-SNAP on SNARE-mediated fusion was reduced in the presence of Mg^2+^ but EDTA, confirming with other groups’ and our previous reconstitution studies that the more free SNAREs recycled from the *cis*-SNARE complex disassembled by NSF and α-SNAP in the fusion system (Fig. 3, *A*–*C*) (26, 32). Next, we sought to determine how membrane fusion kinetics was regulated by Munc18c in the presence of NSF and α-SNAP. We observed that Munc18c significantly enhanced the level of liposome mixing driven by t-and v-SNARE in the presence of NSF and α-SNAP (Fig. 3, *A*–*C*). We observed that the Munc18c-stimulated fusion rate in the reaction containing NSF/α-SNAP/EDTA was lower than that containing NSF/α-SNAP/Mg^2+^ or without NSF/α-SNAP, which might be due to the residual α-SNAP trapped by t-SNAREs. Recent studies reported that the protection function of Munc18-1 against NSF/α-SNAP requires the binding of Munc18-1 to VAMP2 (33). We then studied the effect of v-SNARE on the Munc18c activity by using VAMP8, a non-cognate R-SNARE, to replace VAMP2 in the liposome fusion reactions. We observed that Munc18c could not further promote syntaxin-4/SNAP-23 and VAMP8-driven fusion in the presence of NSF and α-SNAP, suggesting the requirement of cognate v-SNAREs in the fusion stimulation of Munc18c (Fig. 3, *D* and *E*).

It was reported that the membrane fusion might start from SM protein-bound syntaxins, which formed the syntaxin-SM protein-v-SNARE template complexes to chaperone the SNARE assemblies (8, 19, 34, 35, 36, 37). We then examined how Munc18c regulates the non-preassembled syntaxin-4/SNAP-23-mediated membrane fusion in the presence of NSF and α-SNAP. We found that Munc18c dramatically accelerated the non-preassembled t-and v-SNARE-mediated liposome fusion even NSF and α-SNAP were included (Fig. S3). Together, these data demonstrate that Munc18c promotes its cognate GLUT4 SNARE-dependent fusion in the presence of NSF and α-SNAP.

### Munc18c opposes NSF/α-SNAP-mediated *trans*-SNARE disassembly

**Figure 4:**
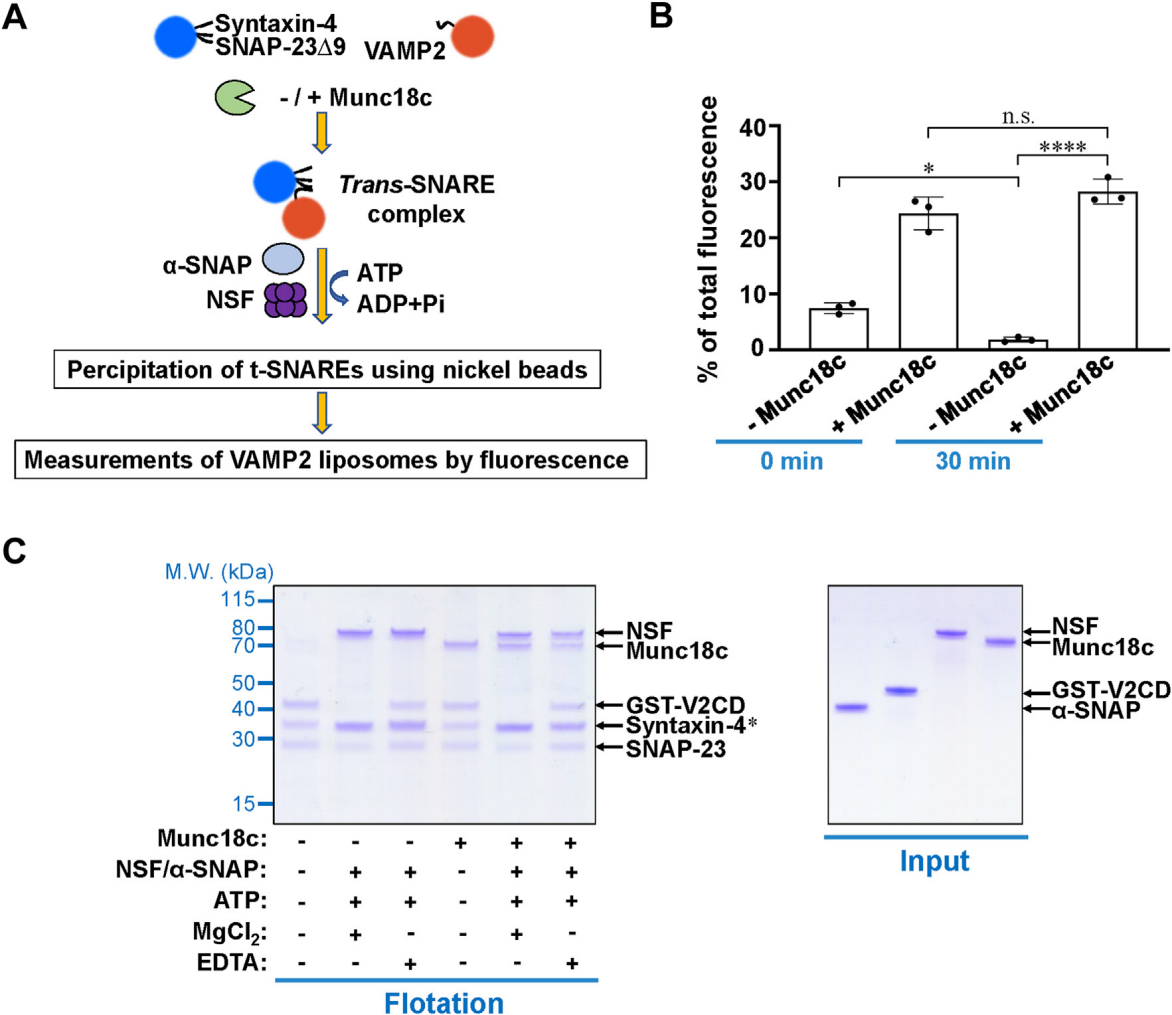
**Munc18c opposesNSF/α-SNAP-mediated *trans*-SNARE disassembly.***A*, diagram of the *trans*-SNARE disassembly assay. *B*, t-SNARE liposomes containing syntaxin-4 and SNAP-23Δ9 were incubated with rhodamine-labeled v-SNARE liposomes in the presence or absence of Munc18c to assemble *trans*-SNARE complexes between membrane bilayers. The *trans*-SNARE disassembly reactions were then performed by incubation with NSF, α-SNAP, and Mg^2+^. Relative amounts of *trans*-SNARE complexes are presented as percentages of maximum rhodamine fluorescence. *p* Values were calculated using ordinary two-way ANOVA with Tukey’s multiple comparisons test. n.s., *p* > 0.05. ∗*p* < 0.05. ∗∗∗∗*p* < 0.0001. *C*, *left*, t-SNARE liposomes containing syntaxin-4 and SNAP-23 were incubated with the GST-tagged cytoplasmic domain of VAMP2 (GST-V2CD) for 1 h at 4 °C to assemble the *cis*-SNARE complex. Liposomes containing *cis*-SNARE complexes were incubated with or without 1 μM NSF, 2 μM α-SNAP, 2.5 mM ATP and 5 mM MgCl_2_/EDTA in the absence or presence of 5 μM Munc18c at 37 °C for 1 h. After flotation on a Nycodenz gradient, proteins bound to the liposomes were resolved on SDS-PAGE and stained with *Coomassie blue*. *Asterisk*, α-SNAP co-migrated with syntaxin-4 on SDS-PAGE. *Right*, *Coomassie blue*-stained SDS-PAGE gel showing the recombinant GST-V2CD, α-SNAP, NSF, and Munc18c proteins used in this study.

NSF and α-SNAP not only disassemble the postfusion cis-SNARE complex, but also interrupt the t-SNARE subcomplex, partially assembled *trans*-SNARE complex, and various misassembled SNARE complexes (38). While NSF and α-SNAP persistently present under physiological conditions, the prefusion *trans*-SNARE complex needs to be protected from the NSF/α-SNAP caused disassembly. Previous studies suggested that some SM proteins, either alone or combined with other factors, could support partially assembled SNARE complexes from being dismantled by NSF/α-SNAP (18, 19, 20, 23, 39). We then established an *in vitro trans*-SNARE complex disassembly assay to explore the function of Munc18c in protecting the *trans*-SNARE complexes from the NSF and α-SNAP- mediated disassembly (Fig. 4*A*). Following the previous studies, we employed a C-terminal truncated SNAP-23 mutant (SNAP-23Δ9), which supports the *trans*-SNARE assembly but could not mediate membrane fusion (40, 41), to prepare the *trans*-SNARE complexes. After the reaction of *trans*-SNARE disassembly with NSF and α-SNAP, the amounts of *trans*-SNARE complexes reduced in the absence of Munc18c. Munc18c efficiently protected the formed *trans*-SNARE complexes between liposomes from disassembly by NSF and α-SNAP (Fig. 4*B*).

We then examined whether the disassembly of the postfusion *cis*-SNARE complex by NSF and α-SNAP can be opposed by Mun18c. Flotation assays showed that the preformed *cis*-SNARE complex was completely disassembled in the presence of NSF and α-SNAP (Fig. 4*C*). When Munc18c was included, the *cis*-SNARE complex was still fully disassembled, suggesting Munc18c opposes NSF/α-SNAP-mediated disassembly of the *trans*-SNARE complex but not the *cis*-SNARE complex.

### SLP is indispensable for Munc18c to overcome the inhibitory activities of α-SNAP and NSF

**Figure 5:**
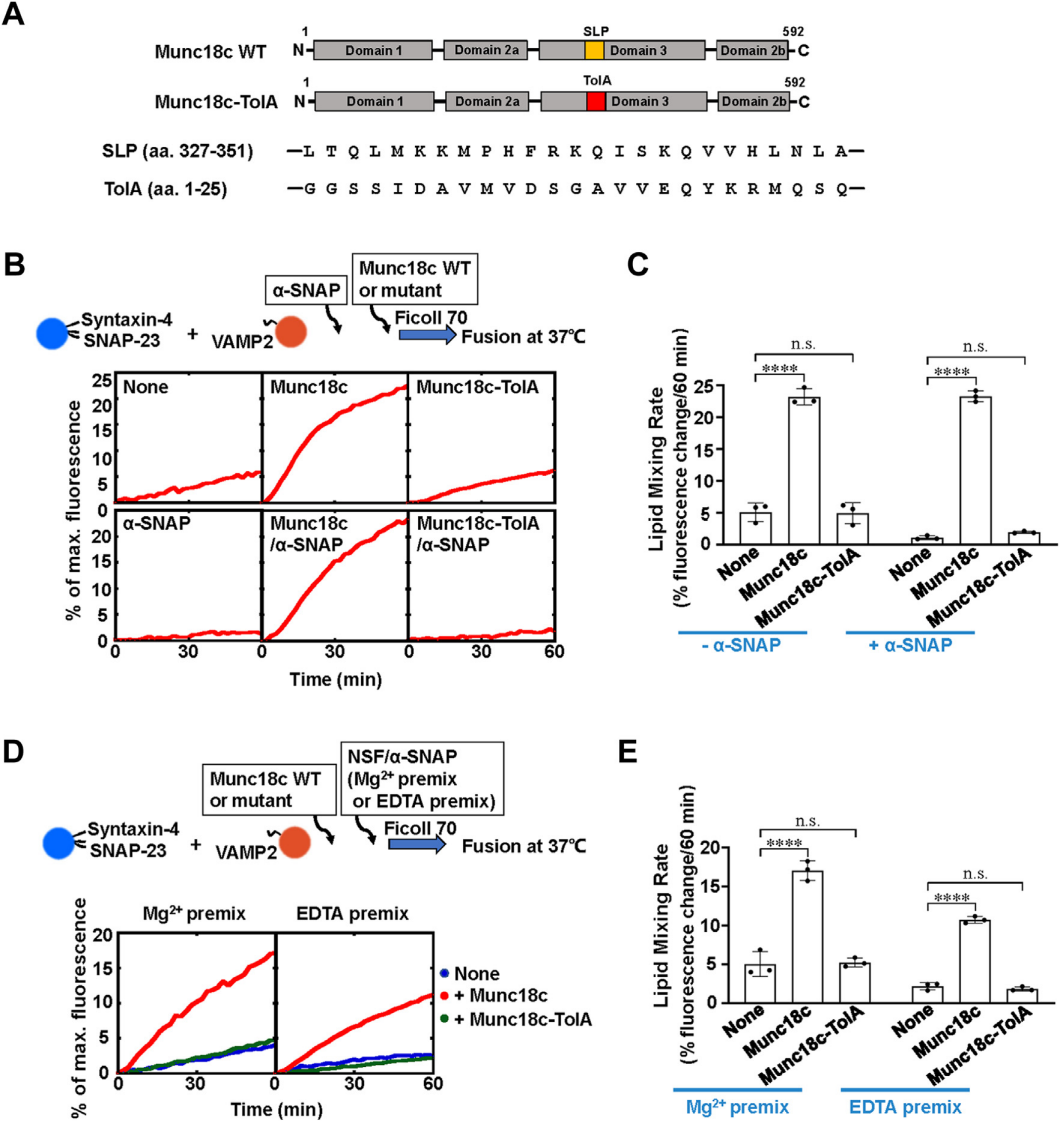
**SLP is essential for Munc18c to promote membrane fusion in the presence of NSF and α-SNAP.***A*, *top*, diagrams of WT and mutant Munc18c proteins. *Bottom*, sequences of SLP and TolA in an equal number of amino acids. *B*, *top*, illustration of the reconstituted liposome fusion procedures. *Bottom*, lipid mixing of recombinant fusion reactions in the presence of 5 μM Munc18c WT or Munc18c-TolA. Each fusion reaction contains 5 μM t-SNAREs, 1.5 μM v-SNARE, 2 μM α-SNAP and 100 mg/ml Ficoll70. *C*, lipid mixing rates of the reconstituted fusion reactions shown in *B*. Data are presented as the percentage of fluorescence change per 60 min. Error bars indicate standard deviation. Data are presented as mean ± SD (n = 3 independent replicates). *p* Values were calculated using ordinary one-way ANOVA with Tukey’s multiple comparisons test. n.s., *p* > 0.05. ∗∗∗∗*p* < 0.0001. *D*, *top*, illustration of the reconstituted liposome fusion procedures. *Bottom*, lipid mixing assays were performed in the presence of 1 μM NSF, 2 μM α-SNAP, 2.5 mM ATP, and 5 mM MgCl_2_ (Mg^2+^ premix)/EDTA (EDTA premix) with 5 μM Munc18c WT or Munc18c-TolA. *E*, lipid mixing rates of the reconstituted fusion reactions shown in *D*. Data are presented as the percentage of fluorescence change per 60 min. Error bars indicate standard deviation. Data are presented as mean ± SD (n = 3 independent replicates). *p* Values were calculated using ordinary two-way ANOVA with Tukey’s multiple comparisons test. n.s., *p* > 0.05. ∗∗∗∗*p* < 0.0001.

We recently showed that Munc18c activates the t-SNAREs to accelerate *trans*-SNARE zippering through the SLP. Next, we explore whether this SLP of Munc18c plays a role in stimulating membrane fusion in the presence of α-SNAP and NSF. In the lipid mixing experiments, the Munc18c-TolA chimeric protein, which was obtained by replacing SLP with an unrelated bacterial helix TolA sequence, could not overcome the inhibition of α-SNAP on SNARE-mediated membrane fusion (Fig. 5, *A*–*C*). Meanwhile, Munc18c-TolA could not accelerate the fusion dynamics in the presence of NSF and α-SNAP (Fig. 5, *D* and *E*), indicating that Munc18c may compete with α-SNAP to bind the t-SNAREs through SLP, excluding α-SNAP and NSF from the fusion SNARE complex. Together, our data suggest that the SLP in domain 3a is essential for Munc18c to promote membrane fusion in the presence of NSF and α-SNAP.

## Discussion

Genetic and biochemical studies have clearly shown that the parallel N-to-C zippering of SNARE complexes *in trans* pulls membranes together to initiate fusion (42, 43, 44), which is accelerated by SNARE regulators like SM proteins (2, 19, 45, 46, 47, 48, 49). α-SNAP binding to the SNAREs results in the recruitment of NSF, prematurely decomposing the *trans*-SNARE complex to block SNARE-mediated membrane fusion (18, 20), raising a question of how *trans*-SNARE complex assembly and membrane fusion proceeds in the presence of NSF and α-SNAP. In addition to being a classic function of NSF recruiting protein, α-SNAP was reported to regulate the assembly of SNARE complex and membrane fusion process independent of NSF (39, 50, 51, 52, 53, 54).

It has been well accepted that SM proteins stimulate *trans*-SNARE complex assembly to stimulate membrane fusion (21, 34, 36, 37, 55). Multiple studies reported that SM protein cooperates with other regulatory factors, for example, Munc13, and synaptotagmin, to promote efficient fusion in the presence of α-SNAP and NSF (18, 19). However, it is still uncertain whether the resistance to α-SNAP and NSF is a conserved function of SM proteins. Munc18c, an SM protein involved in insulin-stimulated GLUT4 translocation, positively regulates GLUT4 SNAREs-dependent vesicle fusion (25, 56, 57). No Munc13s or synaptotagmins involved in GLUT4 translocation make Munc18c an ideal model to study its function in the presence of α-SNAP and NSF. The subtle and dynamic interactions among Munc18c, SNAREs and NSF/α-SNAP may be controlled together to ensure the precise occurrence of fusion. Therefore, it is worth examining how Munc18c regulates its cognate t- and v-SNARE-driven membrane fusion in the presence of NSF and α-SNAP.

Using our reconstituted liposome fusion system, we observed that α-SNAP significantly inhibited SNARE-mediated vesicle fusion. SNAREs and the lipid membrane were both key to the inhibitory effect of α-SNAP, consistent with previous studies (39, 52). Munc18c reversed the inhibition of α-SNAP on SNARE-dependent fusion and significantly increased the fusion rate. Our data suggest that Munc18c competes with α-SNAP in binding to t-SNAREs, and further promotes the assembly of the *trans*-SNARE complex. Munc18c protects *trans*-SNARE complexes rather than *cis*-SNARE complexes from NSF/α-SNAP-driven dismantlement, indicating that Munc18c is topologically sensitive to the recognition and protection of SNARE complex disassembly, promoting efficient SNARE-mediated membrane fusion in the presence of α-SNAP and NSF.

The Vc peptide-mimicking SLP in domain 3a is vital to the fusion stimulatory activity of the SM protein (58, 59). We recently showed that Munc18c SLP interacts with t-SNAREs to promote membrane fusion (22, 27). Here, we discovered that the resistance of Munc18c to α-SNAP and NSF also depends on the SLP. We speculate that there might be a competition between Munc18c SLP and α-SNAP when they interact with t-SNAREs. Munc18c binds to t-SNAREs through SLP, precluding α-SNAP association and facilitating *trans*-SNARE assembly, to simulate SNARE-dependent membrane fusion.

**Figure 6:**
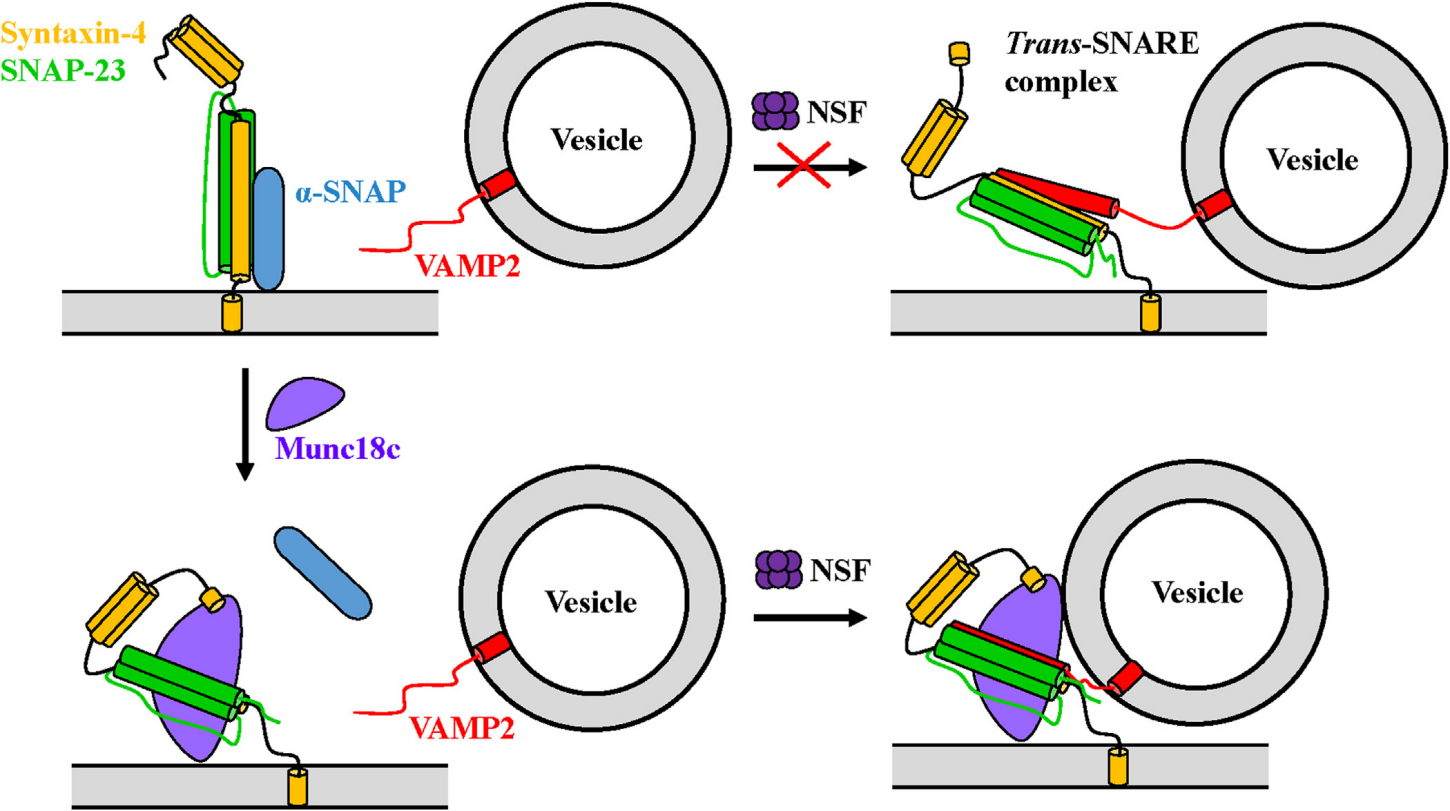
**Model illustrating the mechanism of Munc18c-regulated membrane fusion in the presence of NSF and α-SNAP.** α-SNAP blocks membrane fusion through interactions with t-SNAREs. Munc18c binds to t-SNAREs, precluding α-SNAP association. NSF and α-SNAP destabilize the prefusion t-SNARE subcomplex and *trans*-SNARE complex to avoid the undemanded fusion occurrence. Munc18c recognizes the cognate t- and v-SNARE, protecting prefusion *trans*-SNARE complexes and accelerating SNARE-dependent membrane fusion rate during the specific membrane trafficking pathways.

SNAREs in different pathways have the ability to fuse crossly in the *in vitro* reconstituted system (25, 26), leading to opportunities for incorrect SNARE complex assembly inside the cell, which may cause inappropriate fusion or organelle aggregation. Hence, the accurate spatiotemporal regulation of SNARE-dependent membrane fusion by regulators is highly demanded (8). SNAREs, α-SNAP, NSF, and SM proteins may constitute the dynamic correction system in membrane fusion events. We propose a model whereby Munc18c recognizes the SNARE configuration of specific pathways, protecting prefusion *trans*-SNARE complexes from the disassembly of NSF/α-SNAP and enhancing their fusion activity, while the misassembled or non-cognate SNARE complexes may be eliminated through kinetic proofreading by α-SNAP and NSF (Fig. 6).

In summary, our findings suggest that Munc18c can protect the disassembly of the prefusion *trans*-SNARE complex from NSF/α-SNAP and further accelerate SNARE-dependent membrane fusion. It might be a conserved function for SM proteins to facilitate the *trans*-SNARE complex to resist NSF/α-SNAP in intracellular vesicle fusion.

## Experimental procedures

### Protein expression and purification

Recombinant t- and v-SNARE proteins were expressed in *E. coli* and purified by affinity chromatography (60). Untagged syntaxin-4 and the His_6_-tagged SNAP-23 were expressed using the same procedure as previously described (25, 26). Full-length and cytoplasmic domain (CD) of VAMP2 were expressed similarly as previously described and contained no extra residues after the tags were proteolytically removed by SUMO protease (25, 26). SNAREs were stored in a buffer containing 25 mM HEPES (pH 7.4), 400 mM KCl, 1% n-octyl-β-D-glucoside, 10% (vol/vol) glycerol, and 0.5 mM Tris (2carboxyethyl) phosphine (TCEP). The GST-tagged VAMP2 CD (GST-VAMP2 CD) was purified by GST affinity chromatography. Recombinant α-SNAP and NSF protein was expressed and purified using a His_6_-SUMO tagging system as previously described for VAMP2 (26, 32). The α-SNAP and SNAP-23 mutants were generated by site-directed mutagenesis and purified similar to WT protein. The soluble proteins were stored in a buffer containing 25 mM HEPES (pH 7.4), 150 mM KCl, 10% (vol/vol) glycerol, and 0.5 mM Tris(2carboxyethyl) phosphine (TCEP).

Recombinant untagged Munc18c protein was produced in Sf9 insect cells using baculovirus infection (25). The full-length mouse Munc18c gene was subcloned into the baculovirus transfer vector pFastBac to generate a construct encoding a His_6_-Munc18c fusion protein separated by a TEV protease cleavage site. Munc18c proteins were purified from the Sf9 cells by nickel affinity chromatography as previously described (25). The His_6_ tag was removed from Munc18c by TEV protease, and the protein was subsequently dialyzed overnight against a storage buffer (25 mM HEPES [pH 7.4], 150 mM KCl, 10% glycerol and 0.5 mM TCEP). Munc18c-TolA mutant was generated by site-directed mutagenesis and purified similarly to WT protein (27).

### Proteoliposome preparation

All lipids used in this work were acquired from Avanti Polar Lipids. For t-SNARE reconstitution, 1-palmitoyl-2-oleoyl-sn-glycero-3phosphocholine (POPC), 1-palmitoyl-2-oleoyl-sn-glycero-3-phosphoethanolamine (POPE), 1-palmitoyl-2-oleoyl-sn-glycero-3-phosphoserine (POPS), and cholesterol were mixed in a molar ratio of 60:20:10:10. To prepare v-SNARE liposomes, POPC, POPE, POPS, cholesterol, N-(7-nitro-2,1,3-benzoxadiazole-4-yl)-1,2-dipalmitoyl phosphatidylethanolamine (NBD-DPPE), and N(Lissamine rhodamine B sulfonyl)-1,2-dipalmitoyl phosphatidylethanolamine (rhodamine-DPPE) were mixed at a molar ratio of 60:17:10:10:1.5:1.5.

The liposomes were prepared by detergent dilution and isolated on a Nycodenz density gradient flotation (22, 29). Detergent was removed by overnight dialysis against the reconstitution buffer [25 mM HEPES (pH 7.4), 100 mM KCl, 10% (vol/vol) glycerol, and 1 mM DTT]. To prepare sulforhodamine-loaded liposomes, SNARE liposomes were reconstituted in the presence of 50 mM sulforhodamine B (25, 27). Free dye was removed by overnight dialysis, followed by liposome flotation on a Nycodenz gradient. The protein/lipid ratio was at 1:200 for v-SNAREs and at 1:500 for t-SNARE liposomes.

### FRET-based lipid- and content-mixing assays

A standard liposome fusion reaction contained 5 μM t SNAREs, 1.5 μM v-SNARE, and 100 mg/ml of Ficoll 70 (22, 26, 27). The fusion reactions were conducted in a 96-well microplate at 37 °C. The fusion reactions were carried out in the reaction buffer [25 mM HEPES (pH 7.4), 50 mM KCl, and 1 mM DTT]. In FRET-based lipid mixing assays, v-SNARE liposomes containing NBD-lipids and rhodamine-lipids were directed to fuse with unlabeled t-SNARE liposomes. An increase in NBD fluorescence at 538 nm (excitation at 460 nm) was measured every 2 minutes in a BioTek Synergy HT microplate reader. At the end of the reaction, 10 μl of 10% 3-[(3-cholamidopropyl)dimethylammonio]-2-hydroxy-1-propanesulfonic acid (CHAPSO) was added to the liposomes. For content mixing assays, unlabeled t-SNARE liposomes were directed to fuse with sulforhodamine B-loaded v-SNARE liposomes. The sulforhodamine B fluorescence at 585 nm (excitation at 565 nm) was measured every 2 min. At the end of the reaction, 10 μl of 10% CHAPSO was added to each sample. Fusion data were presented as the percentage of maximum fluorescence change. Full accounting of statistical significance was included for each dataset based on at least three independent experiments.

### Liposome co-flotation assay

The binding of soluble factors with membranes was examined using a liposome co-flotation assay, as we previously described (22, 25, 61). Soluble proteins were incubated with t-SNARE liposomes containing syntaxin-4 and SNAP-23 at 4 °C with gentle agitation. An equal volume (150 μl) of 80% Nycodenz (wt/vol) in the reconstitution buffer was added after 1 h, and the mixture was transferred to 5 mm by 41 mm centrifuge tubes. The liposomes were overlaid with 200 μl each of 35% and 30% Nycodenz and then with 20 μl of reconstitution buffer on the top. The gradients were centrifuged for 4 h at 48,000 rpm in a Beckman SW55 rotor. Liposome samples were collected from the 0/30% Nycodenz interface (2 × 20 μl) and analyzed by SDS-PAGE.

### *Trans*-SNARE disassembly assay

To prepare the *trans*-SNARE complex, t-SNARE liposomes containing syntaxin-4 and SNAP-23Δ9 were incubated with rhodamine-labeled v-SNARE liposomes in the presence or absence of 5 μM Munc18c (40, 41). A 10-fold excess amount of GST-VAMP2 CD was added to block the t-SNARE liposomes without forming the fully assembled *trans*-SNARE complexes, which were resistant to VAMP2 CD treatment (22, 25, 28). 1 μM NSF, 2 μM α-SNAP, 2.5 mM ATP, and 5 mM MgCl_2_ were added into the reactions containing the *trans*-SNARE complexes and incubated for indicated periods at 37 °C. The disassembly was terminated with 5 mM EDTA. The t-SNARE liposomes and bound v-SNARE liposomes were pulled down using nickel beads (through binding to His_6_-SNAP-23). After washing three times with reconstitution buffer, CHAPS was added to the final concentration of 1% to dissolve the bead-bound liposomes. After centrifugation, rhodamine fluorescence in the supernatant was measured in a BioTek Synergy HT microplate reader. v-SNARE liposomes were replaced with protein-free liposomes in a negative control reaction. After subtraction of background fluorescence, the rhodamine fluorescence was used to reflect the relative amounts of *trans*-SNARE complexes (28). The data were presented as percentage of total rhodamine fluorescence of input v-SNARE liposomes. All reactions were performed in the presence of 100 mg/ml Ficoll 70.

### Statistical analysis

All data were presented as the mean ± SD and were analyzed using GraphPad Prism 8.0.2 software for Windows. Statistical significance was calculated using one-way ANOVA or two-way ANOVA, and *p*-Value < 0.05 was considered statistically significant.

## Data availability

All data presented are contained within the main manuscript and supporting information.

## Supporting information

This article contains supporting information.

## Conflict of interest

The authors declare that they have no conflicts of interest with the contents of this article.

## References

[1] Cui L., Li H., Xi Y., Hu Q., Liu H., Fan J. (2022). Vesicle trafficking and vesicle fusion: mechanisms, biological functions, and their implications for potential disease therapy. Mol. Biomed..

[2] Carr C.M., Rizo J. (2010). At the junction of SNARE and SM protein function. Curr. Opin. Cell Biol..

[3] Jahn R., Lang T., Sudhof T.C. (2003). Membrane fusion. Cell.

[4] Sudhof T.C., Rothman J.E. (2009). Membrane fusion: grappling with SNARE and SM proteins. Science.

[5] Jahn R., Scheller R.H. (2006). SNAREs--engines for membrane fusion. Nat. Rev. Mol. Cell Biol..

[6] Wang T., Li L., Hong W. (2017). SNARE proteins in membrane trafficking. Traffic.

[7] Burgoyne R.D., Morgan A. (2007). Membrane trafficking: three steps to fusion. Curr. Biol..

[8] Zhang Y., Hughson F.M. (2021). Chaperoning SNARE folding and assembly. Annu. Rev. Biochem..

[9] Pobbati A.V., Stein A., Fasshauer D. (2006). N- to C-terminal SNARE complex assembly promotes rapid membrane fusion. Science.

[10] Sorensen J.B., Wiederhold K., Muller E.M., Milosevic I., Nagy G., de Groot B.L. (2006). Sequential N- to C-terminal SNARE complex assembly drives priming and fusion of secretory vesicles. EMBO J..

[11] Li F., Pincet F., Perez E., Eng W.S., Melia T.J., Rothman J.E. (2007). Energetics and dynamics of SNAREpin folding across lipid bilayers. Nat. Struct. Mol. Biol..

[12] Barnard R.J., Morgan A., Burgoyne R.D. (1996). Domains of alpha-SNAP required for the stimulation of exocytosis and for N-ethylmalemide-sensitive fusion protein (NSF) binding and activation. Mol. Biol. Cell.

[13] Barnard R.J., Morgan A., Burgoyne R.D. (1997). Stimulation of NSF ATPase activity by alpha-SNAP is required for SNARE complex disassembly and exocytosis. J. Cell Biol..

[14] Barszczewski M., Chua J.J., Stein A., Winter U., Heintzmann R., Zilly F.E. (2008). A novel site of action for alpha-SNAP in the SNARE conformational cycle controlling membrane fusion. Mol. Biol. Cell.

[15] Brunger A.T., Choi U.B., Lai Y., Leitz J., Zhou Q. (2018). Molecular mechanisms of fast neurotransmitter release. Annu. Rev. Biophys..

[16] Song H., Orr A., Duan M., Merz A.J., Wickner W. (2017). Sec17/Sec18 act twice, enhancing membrane fusion and then disassembling cis-SNARE complexes. Elife.

[17] Tomes C.N., De Blas G.A., Michaut M.A., Farre E.V., Cherhitin O., Visconti P.E. (2005). alpha-SNAP and NSF are required in a priming step during the human sperm acrosome reaction. Mol. Hum. Reprod..

[18] Prinslow E.A., Stepien K.P., Pan Y.Z., Xu J., Rizo J. (2019). Multiple factors maintain assembled trans-SNARE complexes in the presence of NSF and alphaSNAP. Elife.

[19] Ma C., Su L., Seven A.B., Xu Y., Rizo J. (2013). Reconstitution of the vital functions of Munc18 and Munc13 in neurotransmitter release. Science.

[20] Xu H., Jun Y., Thompson J., Yates J., Wickner W. (2010). HOPS prevents the disassembly of trans-SNARE complexes by Sec17p/Sec18p during membrane fusion. EMBO J..

[21] Zhang Y., Ma L., Bao H. (2022). Energetics, kinetics, and pathways of SNARE assembly in membrane fusion. Crit. Rev. Biochem. Mol. Biol..

[22] Yu H., Shen C., Liu Y., Menasche B.L., Ouyang Y., Stowell M.H.B. (2018). SNARE zippering requires activation by SNARE-like peptides in Sec1/Munc18 proteins. Proc. Natl. Acad. Sci. U. S. A..

[23] Lobingier B.T., Nickerson D.P., Lo S.Y., Merz A.J. (2014). SM proteins Sly1 and Vps33 co-assemble with Sec17 and SNARE complexes to oppose SNARE disassembly by Sec18. Elife.

[24] Wang S., Li Y., Gong J., Ye S., Yang X., Zhang R. (2019). Munc18 and Munc13 serve as a functional template to orchestrate neuronal SNARE complex assembly. Nat. Commun..

[25] Yu H., Rathore S.S., Lopez J.A., Davis E.M., James D.E., Martin J.L. (2013). Comparative studies of Munc18c and Munc18-1 reveal conserved and divergent mechanisms of Sec1/Munc18 proteins. Proc. Natl. Acad. Sci. U. S. A..

[26] Yu H., Rathore S.S., Shen C., Liu Y., Ouyang Y., Stowell M.H. (2015). Reconstituting intracellular vesicle fusion reactions: the essential role of macromolecular crowding. J. Am. Chem. Soc..

[27] Liu F., He R., Zhu M., Zhou L., Liu Y., Yu H. (2022). Assembly-promoting protein Munc18c stimulates SNARE-dependent membrane fusion through its SNARE-like peptide. J. Biol. Chem..

[28] Liu Y., Wan C., Rathore S.S., Stowell M.H.B., Yu H., Shen J. (2021). SNARE zippering is suppressed by a conformational constraint that is removed by v-SNARE splitting. Cell Rep..

[29] Shen J., Rathore S.S., Khandan L., Rothman J.E. (2010). SNARE bundle and syntaxin N-peptide constitute a minimal complement for Munc18-1 activation of membrane fusion. J. Cell Biol..

[30] Winter U., Chen X., Fasshauer D. (2009). A conserved membrane attachment site in alpha-SNAP facilitates N-ethylmaleimide-sensitive factor (NSF)-driven SNARE complex disassembly. J. Biol. Chem..

[31] Zhou Q., Huang X., Sun S., Li X., Wang H.W., Sui S.F. (2015). Cryo-EM structure of SNAP-SNARE assembly in 20S particle. Cell Res..

[32] Wang S., Liu Y., Crisman L., Wan C., Miller J., Yu H. (2020). Genetic evidence for an inhibitory role of tomosyn in insulin-stimulated GLUT4 exocytosis. Traffic.

[33] Xu Y., Zhu L., Wang S., Ma C. (2022). Munc18 - Munc13-dependent pathway of SNARE complex assembly is resistant to NSF and α-SNAP. FEBS J..

[34] Baker R.W., Jeffrey P.D., Zick M., Phillips B.P., Wickner W.T., Hughson F.M. (2015). A direct role for the Sec1/Munc18-family protein Vps33 as a template for SNARE assembly. Science.

[35] Jiao J., He M., Port S.A., Baker R.W., Xu Y., Qu H. (2018). Munc18-1 catalyzes neuronal SNARE assembly by templating SNARE association. Elife.

[36] Stepien K.P., Xu J., Zhang X., Bai X.C., Rizo J. (2022). SNARE assembly enlightened by cryo-EM structures of a synaptobrevin-Munc18-1-syntaxin-1 complex. Sci. Adv..

[37] Yang J., Jin H., Liu Y., Guo Y., Zhang Y. (2022). A dynamic template complex mediates Munc18-chaperoned SNARE assembly. Proc. Natl. Acad. Sci. U. S. A..

[38] Choi U.B., Zhao M., White K.I., Pfuetzner R.A., Esquivies L., Zhou Q. (2018). NSF-mediated disassembly of on- and off-pathway SNARE complexes and inhibition by complexin. Elife.

[39] Stepien K.P., Prinslow E.A., Rizo J. (2019). Munc18-1 is crucial to overcome the inhibition of synaptic vesicle fusion by alphaSNAP. Nat. Commun..

[40] Lu B. (2015). The destructive effect of botulinum neurotoxins on the SNARE protein: SNAP-25 and synaptic membrane fusion. PeerJ.

[41] Li Y., Wang S., Li T., Zhu L., Ma C. (2018). Tomosyn guides SNARE complex formation in coordination with Munc18 and Munc13. FEBS Lett..

[42] Hanson P.I., Roth R., Morisaki H., Jahn R., Heuser J.E. (1997). Structure and conformational changes in NSF and its membrane receptor complexes visualized by quick-freeze/deep-etch electron microscopy. Cell.

[43] Nichols B.J., Ungermann C., Pelham H.R., Wickner W.T., Haas A. (1997). Homotypic vacuolar fusion mediated by t- and v-SNAREs. Nature.

[44] Weber T., Zemelman B.V., McNew J.A., Westermann B., Gmachl M., Parlati F. (1998). SNAREpins: minimal machinery for membrane fusion. Cell.

[45] Archbold J.K., Whitten A.E., Hu S.H., Collins B.M., Martin J.L. (2014). SNARE-ing the structures of Sec1/Munc18 proteins. Curr. Opin. Struct. Biol..

[46] Baker R.W., Hughson F.M. (2016). Chaperoning SNARE assembly and disassembly. Nat. Rev. Mol. Cell Biol..

[47] Carpp L.N., Ciufo L.F., Shanks S.G., Boyd A., Bryant N.J. (2006). The Sec1p/Munc18 protein Vps45p binds its cognate SNARE proteins via two distinct modes. J. Cell Biol..

[48] Demircioglu F.E., Burkhardt P., Fasshauer D. (2014). The SM protein Sly1 accelerates assembly of the ER-Golgi SNARE complex. Proc. Natl. Acad. Sci. U. S. A..

[49] Diao J., Su Z., Lu X., Yoon T.Y., Shin Y.K., Ha T. (2010). Single-vesicle fusion assay reveals Munc18-1 binding to the SNARE core is sufficient for stimulating membrane fusion. ACS Chem. Neurosci..

[50] Rodriguez F., Bustos M.A., Zanetti M.N., Ruete M.C., Mayorga L.S., Tomes C.N. (2011). alpha-SNAP prevents docking of the acrosome during sperm exocytosis because it sequesters monomeric syntaxin. PLoS One.

[51] Ma L., Kang Y., Jiao J., Rebane A.A., Cha H.K., Xi Z. (2016). Alpha-SNAP enhances SNARE zippering by stabilizing the SNARE four-helix bundle. Cell Rep..

[52] Park Y., Vennekate W., Yavuz H., Preobraschenski J., Hernandez J.M., Riedel D. (2014). alpha-SNAP interferes with the zippering of the SNARE protein membrane fusion machinery. J. Biol. Chem..

[53] Schwartz M.L., Nickerson D.P., Lobingier B.T., Plemel R.L., Duan M., Angers C.G. (2017). Sec17 (alpha-SNAP) and an SM-tethering complex regulate the outcome of SNARE zippering *in vitro* and *in vivo*. Elife.

[54] Zick M., Orr A., Schwartz M.L., Merz A.J., Wickner W.T. (2015). Sec17 can trigger fusion of trans-SNARE paired membranes without Sec18. Proc. Natl. Acad. Sci. U. S. A..

[55] Yu H., Shen J. (2023). Faithful SM proteins chaperone SNAREs on path to successful assembly. Proc. Natl. Acad. Sci. U. S. A..

[56] Oh E., Spurlin B.A., Pessin J.E., Thurmond D.C. (2005). Munc18c heterozygous knockout mice display increased susceptibility for severe glucose intolerance. Diabetes.

[57] Dolai S., Liang T., Orabi A.I., Xie L., Holmyard D., Javed T.A. (2018). Depletion of the membrane-fusion regulator Munc18c attenuates caerulein hyperstimulation-induced pancreatitis. J. Biol. Chem..

[58] Zhang X., Rebane A.A., Ma L., Li F., Jiao J., Qu H. (2016). Stability, folding dynamics, and long-range conformational transition of the synaptic t-SNARE complex. Proc. Natl. Acad. Sci. U. S. A..

[59] Melia T.J., Weber T., McNew J.A., Fisher L.E., Johnston R.J., Parlati F. (2002). Regulation of membrane fusion by the membrane-proximal coil of the t-SNARE during zippering of SNAREpins. J. Cell Biol..

[60] Shen J., Tareste D.C., Paumet F., Rothman J.E., Melia T.J. (2007). Selective activation of cognate SNAREpins by Sec1/Munc18 proteins. Cell.

[61] He R., Liu F., Wang H., Huang S., Xu K., Zhang C. (2023). ORP9 and ORP10 form a heterocomplex to transfer phosphatidylinositol 4-phosphate at ER-TGN contact sites. Cell. Mol. Life Sci..

