# Supplementary material for: Munc18c accelerates SNARE-dependent membrane fusion in the presence of regulatory proteins α-SNAP and NSF
Source: J Biol Chem. 2024 Feb 21;300(3):105782. doi: 10.1016/j.jbc.2024.105782 (PMC10959665; doi:10.1016/j.jbc.2024.105782)
Supplement: Supplemental Figures S1–S3 [file mmc1.pdf]

Munc18c accelerates SNARE-dependent membrane fusion in the presence of regulatory proteins α-SNAP and NSF

Furong Liu[1#], Ruyue He[1#], Xinyu Xu[1#], Min Zhu[1], Haijia Yu[1*], and Yinghui Liu[1*]

[1] Jiangsu Key Laboratory for Molecular and Medical Biotechnology, College of Life Sciences, Nanjing Normal University, Nanjing, 210023, China.

[#] These authors contributed equally

*Correspondence: yinghuiliu@njnu.edu.cn (Y.L.); yuhaijia@njnu.edu.cn (H.Y.)

This SI file contains: Supplemental Figures 1 to 3.

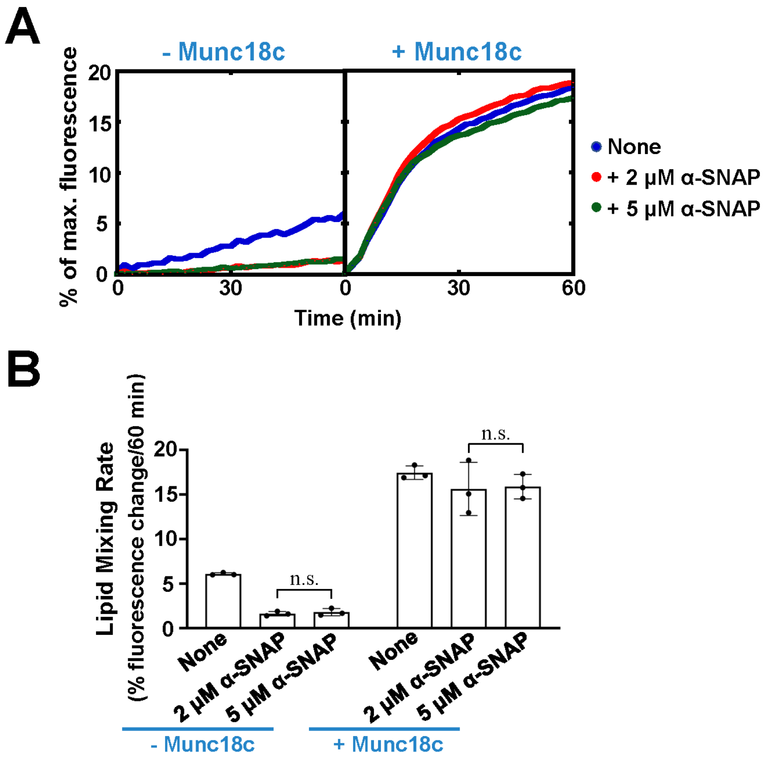

Figure S1. Munc18c stimulates α-SNAP-blocked membrane fusion. *A,* Lipid mixing of the reconstituted fusion reactions in the absence or presence of α-SNAP or Munc18c. Each fusion reaction contained 5 µM t-SNAREs, 1.5 µM v-SNARE and 100 mg/mL Ficoll 70. The concentration of α-SNAP was 2 µM or 5 µM. The concentration of Munc18c was 5 µM. *B,* Lipid mixing rates of the reconstituted fusion reactions shown in *A*. Data are presented as percentage of fluorescence change per 60 min. Error bars indicate standard deviation. Data are presented as mean ± SD (n = 3 independent replicates). p Values were calculated using two-way ANOVA with Tukey's multiple comparisons test. n.s., $p > 0.05$.

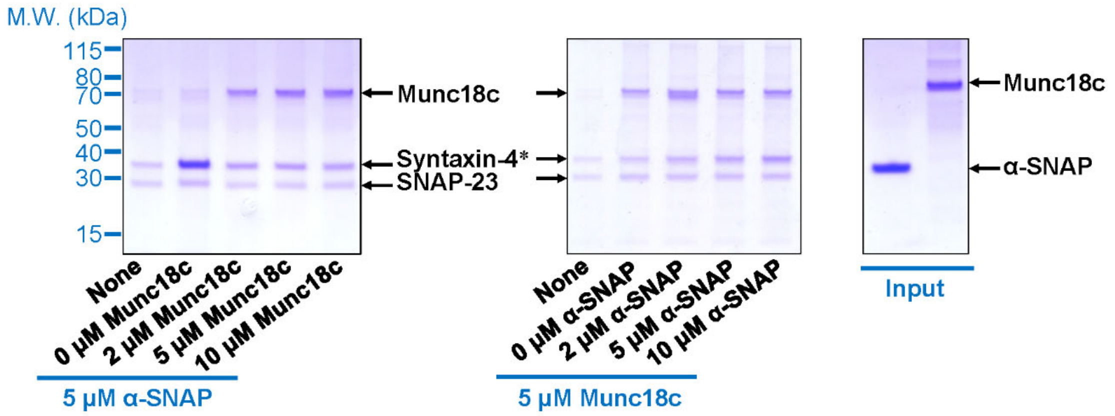

Figure S2. Munc18c efficiently displaced α-SNAP from t-SNAREs. In the competitive binding assays, t-SNARE liposomes containing syntaxin-4 and SNAP-23 were preincubated with 5 μM α-SNAP or Munc18c. Munc18c or α-SNAP was added with the indicated concentrations. Liposome flotation assay was used to monitor the association of proteins with t-SNAREs.

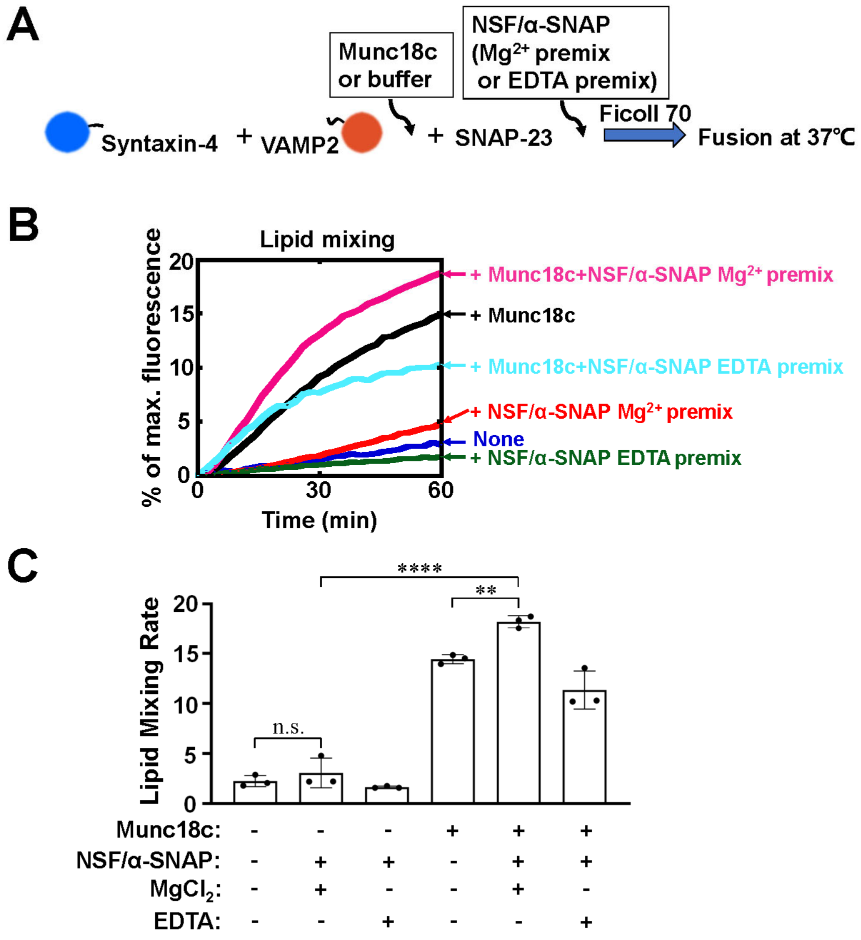

Figure S3. Munc18c promotes non-preassembled SNARE-driven membrane fusion in the presence of NSF and α-SNAP. *A,* Illustration of the experimental procedure of the reconstituted fusion reactions. *B,* Lipid mixing of the reconstituted fusion reactions shown in A. Lipid mixing assays were performed in the presence of 1 µM NSF, 2 µM α-SNAP, 2.5 mM ATP, and 5 mM $MgCl_2$ (Mg^{2+} premix) /EDTA (EDTA premix) without or with 5 µM Munc18c. Each fusion reaction contained 5 µM t-SNAREs, 1.5 µM v-SNARE and 100 mg/mL Ficoll 70. *C,* Lipid mixing rates of the reconstituted fusion reactions shown in *B*. Data are presented as the percentage of fluorescence change per 60 min. Error bars indicate standard deviation. Data are presented as mean ± SD (n = 3 independent replicates). p Values were calculated using two-way ANOVA with Tukey's multiple comparisons test. n.s., $p > 0.05$. **$p < 0.01$. ****$p < 0.0001$.
